# Mesenchymal Stem Cell-Derived Exosomes Inhibit Stim1–Orai1 Signaling and Calcium Overload-Induced Mitochondrial Damage of Follicular Helper T Cells in Lupus

**DOI:** 10.34133/bmr.0255

**Published:** 2025-09-22

**Authors:** Yingyu Wang, Qingyong Xiang, Yueren Wu, Xiaoyun Zhang, Zhongzhou Huang, Yunxia Hou, Yan Wang, Ji Yang, Weiguo Wan, Hejian Zou, Xue Yang

**Affiliations:** ^1^ Division of Rheumatology, Huashan Hospital, Fudan University, Shanghai, China.; ^2^ Institute of Rheumatology, Immunology and Allergy, Fudan University, Shanghai, China.; ^3^ Department of Rheumatology, Shanghai Fifth Peoples Hospital, Fudan University, Shanghai, China.; ^4^ Department of Integrative Medicine, Huashan Hospital, Fudan University, Shanghai, China.; ^5^ Institutes of Integrative Medicine, Fudan University, Shanghai, China.; ^6^ Department of Dermatology, Sun Yat-sen Memorial Hospital, Sun Yat-sen University, Guangzhou, China.; ^7^ Department of Rheumatology and Immunology, The Affiliated Hospital of Inner Mongolia Medical University, Hohhot, Inner Mongolia, China.; ^8^ Central Lab, Huashan Hospital, Fudan University, Shanghai, China.; ^9^ Department of Dermatology, Zhongshan Hospital, Fudan University, Shanghai, China.

## Abstract

Systemic lupus erythematosus (SLE) is an autoimmune disorder characterized by aberrant T cell activity and excessive autoantibody production. Follicular helper T cells (Tfh) play a pivotal role in promoting B cell-mediated autoantibody generation, contributing to SLE progression. Although mesenchymal stem cell-derived exosomes (MSC-Exos) exhibit immunomodulatory properties, their effects on Tfh in SLE and the underlying mechanisms remain unclear. To address this, we first analyzed sorted Tfh from an imiquimod-induced lupus murine model (IMQ-SLE) and found that MSC-Exos effectively suppressed Tfh function. Consistently, Tfh polarization assays demonstrated that MSC-Exos modulate Tfh differentiation in vitro. Subsequently, we evaluated the therapeutic potential of intravenous MSC-Exos administration and confirmed that MSC-Exos markedly inhibited Tfh expansion and function in vivo. Further RNA sequencing followed by validation experiments identified that MSC-Exos restore calcium homeostasis in Tfh. Mechanically, MSC-Exos down-regulate stromal interaction molecule 1 (Stim1) and Orai1 expression, inhibiting nuclear factor of activated T cells (NFAT) and nuclear factor κB (NF-κB) activation. In parallel, MSC-Exos mitigate calcium overload-induced mitochondrial damage by suppressing mitochondrial calcium uniporter (MCU) expression. Finally, we observed that MSC-Exos also promote the differentiation of follicular regulatory T cells (Tfr) both in vivo and in vitro. These findings suggest that MSC-Exos ameliorate SLE by correcting cellular calcium dysregulation and mitochondrial damage in Tfh while simultaneously restoring the Tfh/Tfr imbalance, highlighting their potential as a therapeutic strategy for SLE.

## Introduction

Systemic lupus erythematosus (SLE) is a chronic autoimmune disease characterized by the involvement of multiple organs and the excessive formation of autoantibodies, which is one of its key pathogenic mechanisms. Follicular helper T cells (Tfh), a critical subset of CD4^+^ T cells in lupus, are characterized by high expression of CXCR5, PD-1, interleukin-21 (IL-21), and the specific transcription factor BCL6 [1]. They provide essential signals for the survival, affinity maturation, differentiation, and proliferation of B cells within germinal centers (GCs), facilitating the secretion of high-affinity antibodies [2]. Increased and overactivated Tfh in SLE are positively correlated with disease activity, and our previous study indicated that inhibiting Tfh expansion can significantly alleviate lupus symptoms [3]. While the role of Tfh in SLE is well established, the upstream regulators of their hyperactivity and the targeted intervention remain unexplored.

Stem cell therapy shows promise in treating autoimmune diseases owing to its immunoregulatory capacity. Mesenchymal stem cells (MSCs) effectively mitigate lupus-related inflammation by suppressing T helper 1 (Th1)/Th17 cell activity and promoting regulatory T cell (Treg) differentiation [4,5]. Our prior research demonstrated that bone marrow-derived MSCs suppress Tfh differentiation and alleviate lupus nephritis [3]. However, concerns regarding MSC therapy include potential tumorigenic risks, immune rejection, and unstable production quality control [6]. Compared to MSCs, extracellular exosomes derived from MSCs (MSC-Exos) offer a cell-free therapeutic approach with enhanced biological safety and serve as primary mediators of MSC immunoregulatory functions. Accumulating evidence highlights the systemic anti-inflammatory effects of MSC-Exos across diverse autoimmune conditions. In neurological, gastrointestinal, and respiratory disorders, MSC-Exos effectively drive macrophage polarization toward anti-inflammatory phenotypes, thereby alleviating various autoinflammatory conditions [7–9]. In T cell studies, MSC-Exos restore immune equilibrium by reducing Th1/Th17 populations and expanding Tregs, ameliorating tissue inflammation in inflammatory bowel disease (IBD) and highlighting their potential to regulate T cell-mediated immune responses, although specific mechanisms were not elucidated [10]. Another study reveals that MSC-Exos suppress the activated T cell proliferation through the induction of cell cycle arrest, without specifying the T cell subset involved. Beyond immune cells, emerging mechanistic studies further demonstrate that MSC-Exos exert the anti-inflammatory effect by mitigating oxidative stress, stabilizing mitochondria, and suppressing mitochondrial DNA (mtDNA) release, thereby alleviating intracellular inflammation [11,12]. Regarding lupus studies, MSC-Exos enhance efferocytosis and the anti-inflammatory M2 polarization of macrophages while inducing Treg cells, thus alleviating lupus progression [13]. Despite these advances, critical gaps persist: While current studies predominantly focus on the MSC-Exos-mediated regulation of macrophage polarization, Th17, and Treg cells in lupus, their ability to modulate Tfh—the key drivers of pathogenic autoantibody production—remains entirely unexplored, leaving a key therapeutic opportunity unaddressed.

To bridge this gap, our study systematically investigates MSC-Exos’ regulatory effects on Tfh and their underlying mechanisms. RNA-sequencing (RNA-seq) and bioinformatics analyses reveal that Tfh in SLE exhibit aberrant calcium signaling, which can be modulated by MSC-Exos. By simultaneously addressing cytosolic and mitochondrial calcium dysregulation, MSC-Exos inhibit Tfh hyperactivation in SLE. This dual mechanism not only highlights novel therapeutic targets for Tfh in lupus but also underscores the potential of MSC-Exos in lupus treatment.

The immune dysregulation observed in SLE is not attributed to a single T cell subset but results from the combined imbalance among various T cell subsets. Similar to Th17/Treg imbalance, the Tfh/Tfr imbalance—resulting in excessive immune activation—has emerged as a pivotal contributor to SLE [14]. Tfr, a specialized subset of Treg cells sharing phenotypic similarities with Tfh, exhibit high expression of Foxp3 and IL-10. Through CTLA-4, IL-10, and transforming growth factor-β (TGF-β) secretion, Tfr antagonize Tfh-mediated B cell help, thereby restraining autoantibody production [14]. Conversely, Tfh-derived IL-21 suppresses Foxp3 expression in Tfr [15], creating a self-reinforcing cycle where Tfh hyperproliferation further destabilizes Tfr differentiation. Clinical evidence confirms that elevated Tfh/Tfr ratios in SLE patients correlate with disease severity [14], and strategy addressing this imbalance such as low-dose IL-2 shows promise in disease remission [16]. However, current interventions often lack cell type specificity, underscoring the need for targeted therapies capable of simultaneously correcting Tfh hyperactivity while boosting Tfr function. Addressing this unmet need, our study demonstrates that MSC-Exos exert a bidirectional regulatory effect on the Tfh/Tfr balance, exhibiting MSC-Exos’ unique ability to restore immune homeostasis, opening new doors for SLE therapy.

## Materials and Methods

### MSC cultivation

Adipose-derived MSC (AD-MSC) cell lines, sourced from adult liposuction, were obtained from Oricell (HUXMD-01001, China) and cultured in Dulbecco’s modified Eagle’s medium/F-12 medium (Gibco, 8120254, USA) supplemented with 15% calf serum devoid of exosomes (Umibio, UR50202, China), along with 100 U/ml penicillin and streptomycin (Gibco, 15140122, USA). The cells were seeded at a density of 1 × 10^6^ cells in 10-cm tissue culture dishes (Corning, USA) and maintained at 37 °C in a 5% CO_2_ humidified atmosphere.

### Identification and uptake of MSC-Exos

AD-MSCs were cultured for 48 to 72 h until cell fusion reached approximately 80% to 90%, after which the supernatants were collected for exosome extraction. Only MSCs within 5 passages were used for exosome extraction. Exosomes were isolated through differential centrifugation to remove cells and debris, followed by ultracentrifugation at 100,000*g* for 60 min at 4 °C using a Beckman Coulter superhigh-speed centrifuge (Optima XPN-100, USA). The exosomal pellets were washed in phosphate-buffered saline (PBS), centrifuged again at 100,000*g* for 60 min at 4 °C, and then resuspended in PBS before storage at −80 °C. The protein concentration of MSC-Exos was determined using a bicinchoninic acid (BCA) protein assay kit (Thermo, A55861, USA), while the size distribution and particle concentration were assessed using a Nanosight NS300 instrument (Malvern Instruments, Worcestershire, UK) (Fig. S1). Transmission electron microscopy (TEM) (Tecnai G2 Spirit, FEI, USA) was utilized to examine the morphology of AD-MSC exosomes (MSC-Exos) (Fig. S2), and Western blotting (WB) confirmed the expression of surface markers such as CD63 and Alix, and the negative marker Histone 3 was also verified (Fig. S3).

### SLE patients

For this study, 28 patients diagnosed with SLE meeting the criteria set forth by the Revised Criteria of the American College of Rheumatology and the 2019 European League Against Rheumatism/American College of Rheumatology (EULAR/ACR) classification criteria for SLE were enrolled. Ethical approval was obtained from the Ethics Committee of Zhongshan Hospital, Fudan University, Shanghai, China (approval no. B2023-0862R), and written informed consent was obtained from all participants. The study adhered to the principles outlined in the Helsinki Declaration, and all participants provided both written and verbal informed consent prior to participation.

### Mice

Specific pathogen-free (SPF) Balb/C mice (weight, 18 to 22 g; age, 6 weeks) were obtained from the Animal Experiment Center of Fudan University (Shanghai, China) and housed in an SPF colony at the Animal Center of Shanghai Medical School, Fudan University. All experimental procedures conducted on the mice followed the National Institutes of Health Guidelines on Laboratory Research and were approved by the Animal Care Committee of Huashan Hospital (approval 2023-HSYY-174JZS).

### IMQ-SLE model construction

Mice were randomly divided into 3 groups: imiquimod (IMQ)-SLE with PBS injection, IMQ-SLE with MSC-Exos injection, and WT (wild-type) groups. Six-week-old WT mice were subjected to IMQ treatment according to established protocols to induce a lupus-like phenotype [17,18]. Specifically, 5% IMQ cream was applied topically to the inner ear 3 times a week for 10 weeks. Mice were sacrificed 14 d after injection. Spleens were isolated and mechanically dissociated through a 70-μm sterile filter to obtain single-cell suspensions.

### Isolation of Tfh in IMQ-SLE

Tfh were sorted using EasySep Naive CD4^+^ T cell Isolation Kit (Stemcell, 19765, CA), allophycocyanin (APC)-conjugated anti-CXCR5 (Invitrogen, 17-7185-82, USA), and EasySep APC Positive Selection Kit II (Stemcell, 17667, CA). The purity of sorted CD4^+^ T cells was greater than 98% (Fig. S4), and the purity of sorted CXCR5^+^ T cells was greater than 60%. Sorted naive CD4^+^ T cells were suspended in RPMI 1640 medium (GIBCO, 11875093, USA) supplemented with 100 U/ml penicillin, 100 mg/ml streptomycin, 0.05 mM β-mercaptoethanol, and 15% fetal bovine serum (Hyclone, SH30070, CA).

### Induction of T follicular helper cell differentiation in vitro

Naive CD4^+^ T cells were isolated from the spleens of Balb/C mice using the EasySep Naive CD4^+^ T Cell Isolation Kit (Stemcell, 19765, CA), achieving a purity exceeding 98% (Fig. S5). These sorted cells were suspended in RPMI 1640 medium (GIBCO, 11875093, USA) supplemented with 100 U/ml penicillin, 100 mg/ml streptomycin, 0.05 mM β-mercaptoethanol, and 15% fetal bovine serum (Hyclone, SH30070, CA). For Tfh polarization, the medium was additionally supplemented with 20 ng/ml IL-6, 10 μg/ml anti-interferon-γ (IFN-γ), 10 μg/ml anti-IL-4, 10 μg/ml anti-IL-2, and 10 μg/ml anti-TGF-β (PeproTech-BioGems, USA). Cell suspensions containing 2 × 10^6^ cells were then seeded onto a 96-well plate precoated with 8 mg/ml anti-CD3 (PeproTech-BioGems, 05112-25-500, USA) and 8 mg/ml anti-CD28 (PeproTech-BioGems, 10312-25-500, USA) (Fig. S6). Cells were cultured in a 5% CO_2_ atmosphere for 72 h before analysis.

### Flow cytometry analysis

Flow cytometry was used to evaluate the expression of surface markers and intracellular molecules using BD FACSCanto II and FACSAria III flow cytometers (BD Biosciences, NJ, USA). For intracellular cytokine staining, cells were stimulated for 6 h with Cell Stimulation Cocktail Plus Protein Transport Inhibitors (ESscience Biotech, ECS005, ECS006, China). Intracellular staining of BCL6, IL-21, IL-10, and Foxp3 was performed using a Foxp3 staining kit (eBioscience, 2258710, USA). The following antibodies were used: Zombie Green Fixable Viability Kit (BioLegend, 423111, USA), APC-conjugated anti-CXCR5 (BioLegend, 145506, USA), phycoerythrin (PE)-conjugated anti-PD-1 (BioLegend, 135253, USA), PE/cyanine7-conjugated anti-PD-1 (BioLegend, 135215, USA), PE-conjugated Bcl-6 (BioLegend, 358504, USA), and PE/cyanine7-conjugated Foxp3 (Invitrogen, 2833598, USA). Data analysis was carried out using FlowJo software.

### Real-time quantitative polymerase chain reaction analysis

Total RNA was extracted from the cells using the RNA-Quick Purification Kit (ESscience Biotech, RN001, China) following the manufacturer’s protocol. Total RNA (1 μg) was reverse-transcribed into cDNA in a 10-μl reaction volume using the PrimeScript RT Reagent Kit (Takara, RR037A, Japan). The relative expression levels of BCL6, IL-21, CXCR5, IL-10, Foxp3, CaN, NFATc1, NFATc2, Orai1, Stim1, and Stim2 were assessed using real-time quantitative polymerase chain reaction (RT-qPCR) at the RNA level. For PCR amplification, 1.5 μl of cDNA was used as the template. The QuantStudio 6 RT-qPCR instrument (Thermo, USA) facilitated the reaction with a cycling program of 95 °C for 60 s, followed by 40 cycles of 95 °C for 15 s and 60 °C for 34 s. Each sample was run in triplicate and normalized to the level of β-actin. The relative mRNA levels were determined using the 2−ΔΔCt method and represented as fold change compared with the control group. The primer sequences used for mRNA amplification are provided in Table S1.

### WB analysis

Cell lysates were prepared by treating cells with cell lysis buffer (Beyotime, P0013C, China) supplemented with protease and phosphatase inhibitors (Beyotime, P1045, China). The supernatant, collected after centrifugation at 12,000*g* for 20 min at 4 °C, was used to determine protein concentration using a BCA protein assay kit (Thermo, A55861, USA). Proteins were then separated by sodium dodecyl sulfate–polyacrylamide gel electrophoresis and transferred onto polyvinylidene fluoride membranes. These membranes were blocked with 5% nonfat milk at 25 °C for 1 h. Primary antibodies targeting CaN (Proteintech, 13422-1-AP, China), NFATc2 (Proteintech, 22023-1-AP, China), P65 (Affinity, AF5006, USA), p-P65 (Affinity, AF2006, USA), IκB (Abmart, T55026, China), p-IκB (Abmart, TA2002, China), Stim1 (Proteintech, 11565-1-AP, China), Orai1 (Proteintech, 28411-1-AP, China), and MCU (Abways, BY0101, China) were incubated overnight at 4 °C. Chemiluminescence detection was performed using enhanced chemiluminescence reagents, and images were captured using film cassette exposure (TANON Science & Technology, China).

### ELISA analysis

Enzyme-linked immunosorbent assay (ELISA) assays were conducted using the following kits: the Mouse anti-double-stranded DNA (dsDNA) immunoglobulin G (IgG)-specific ELISA Kit (Alpha Diagnostic International, 5120, USA), the Mouse Anti-Nuclear Antigens (ANA/ENA) Ig’s (total IgA, IgG, and IgM) ELISA Kit (Alpha Diagnostic International, 5210, USA), and the Mouse Complement Component 3 (C3) ELISA Kit (Alpha Diagnostic International, 6270, USA). All procedures were performed according to the manufacturer’s instructions.

### Measurement of the Ca^2+^ concentration

The following probes were used: Fluo-4 AM (YEASEN, 40704ES50, China) and Rhod-2 AM (YEASEN, 40776ES50, China). Intracellular Ca^2+^ concentration was measured using a Ca^2+^ probe Fluo-4 AM according to the manufacturer’s protocol. Mitochondrial Ca^2+^ were monitored in live cells using Rhod-2 AM according to the manufacturer’s protocol. Tfh were loaded with 4 μM Fluo-4 AM or Rhod-2 AM in serum-free medium for 30 min at 37 °C, followed by washes with Hanks’ balanced salt solution (HBSS) buffer to remove excess dye. After washing, the cells were incubated with HBSS for 30 min before testing. Flow cytometry was used to evaluate the Ca^2+^ concentration using BD FACSCanto II (BD Biosciences, NJ, USA), with Fluo-4 exhibiting increased green fluorescence intensity in positive cells, and Rhod-2 exhibiting increased red fluorescence intensity in positive cells. Data analysis was carried out using FlowJo software.

### Measurement of the ROS, MMP, and mPTP

Intracellular reactive oxygen species (ROS) levels were measured by DCFH-DA (Beyotime, S0033S, China). Mitochondrial membrane potential (MMP, ΔΨm) was measured by JC-1 assay kit (Beyotime, C2006, China). Mitochondrial permeability transition pore (mPTP) opening levels were measured by calcein (Abbkine, BMD0064, China). Tfh were stained with DCFH-DA, JC-1, or calcein according to their manufacturer’s manual and analyzed by BD FACSCanto II (BD Biosciences, NJ, USA), respectively.

### RNA sequencing

Naïve CD4^+^ T cells were collected from 3 WT, then cultured for 72 h under Tfh polarizing conditions, and intervened with MSC-Exos (*n* = 3) or PBS (*n* = 3). Three sets of paired cell samples were collected for RNA-seq. The data that support the findings of this study have been deposited into CNGB Sequence Archive (CNSA) [19] of China National GeneBank DataBase (CNGBdb) with accession number CNP0006973. All relevant data are available upon reasonable request.

### TEM analysis

Cells were fixed with TEM fixer (Solarbio, P1126, China) at 4 °C for 12 h, followed by pre-embedding in 1% agarose and immobilization in 1% osmium tetroxide. After ethanol dehydration at room temperature, samples were embedded in Poly/Bed 812 resin and polymerized at 65 °C. Ultrathin sections were stained with 2% uranyl acetate and 2.6% lead citrate, and analyzed using a transmission electron microscope (Hitachi, Japan).

### Statistical analysis

Statistical data were expressed as mean ± standard error of the mean. A two-tailed unpaired Student’s *t* test was used to evaluate differences between two groups. Multiple comparisons were conducted using one-way analysis of variance (ANOVA) with Tukey’s multiple comparisons test. Statistical significance was determined at a *P* value of <0.05. All statistical analyses were performed using GraphPad Prism version 8.0 (GraphPad Software, USA).

## Results

### Tfh expansion accompanied by Tfh/Tfr imbalance in lupus

**Fig. 1.**
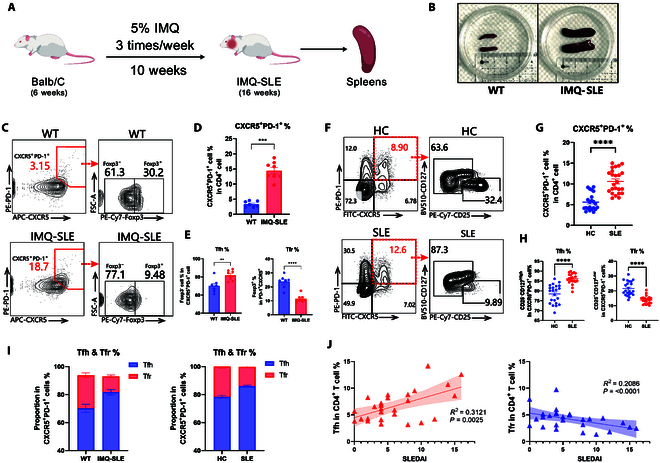
The imbalance of Tfh/Tfr in IMQ-SLE mice and lupus patients. (A) Establishment process of the IMQ-SLE model. (B) Comparison of spleen sizes between WT and IMQ-SLE. (C) Murine CXCR5^+^PD-1^+^ cells were gated on CD4^+^ cells, and Foxp3^−^ Tfh and Foxp3^+^ Tfr cells were gated on CXCR5^+^PD-1^+^ cells in WT and IMQ-SLE mice. (D) Splenic CD4^+^CXCR5^+^PD-1^+^ frequencies in IMQ-SLE mice (*n* = 8) versus WT (*n* = 7). (E) Proportions of Foxp3^−^ Tfh and Foxp3^+^ Tfr gated on CXCR5^+^PD-1^+^ cells in WT (*n* = 7) versus IMQ-SLE (*n* = 8) mice. (F) Human CXCR5^+^PD-1^+^ cells were gated on CD4^+^ cells, and Tfh (CD25^−^CD127^High^) and Tfr (CD25^+^CD127^Low^) cells were gated on CXCR5^+^PD-1^+^ cells in healthy controls (HC) and SLE patients. (G) Peripheral CXCR5^+^PD-1^+^ cell frequencies in HC (*n* = 24) versus SLE patients (*n* = 23). (H) Proportions of CD25^−^CD127^High^ Tfh and CD25^+^CD127^Low^ Tfr in HC (*n* = 24) versus SLE patients (*n* = 23). (I) Tfh accumulation and Tfr depletion in SLE mice and patients. (J) Frequencies of Tfh and Tfr and their correlation in patients with different SLEDAI scores. Bars indicate the means ± SEM. *P < 0.05, **P < 0.01, ***P < 0.001, ****P < 0.0001 determined by 2-tailed unpaired Student’s *t* test.

The lupus mouse model induced by the Toll-like receptor 7 (TLR7) agonist IMQ effectively mimics various clinical manifestations of lupus [17,18]. In our study, we administered IMQ to 6-week-old female Balb/C mice over a 10-week period (Fig. 1A). Post-induction, these IMQ-treated mice exhibited significant splenomegaly (Fig. 1B). Tfh were identified by surface markers, including high expression of CXCR5 and PD-1, along with significant levels of intracellular BCL6 and IL-21. Flow cytometry analysis revealed a significant increase in the proportion of CD4^+^CXCR5^+^PD-1^+^ cells in the spleens of IMQ-SLE mice compared to WT controls (Fig. 1C and D). Tfr shared similar surface markers with Tfh but also expressed Foxp3; thus, we also investigated the frequency of Tfr simultaneously. Conversely, the proportion of CD4^+^CXCR5^+^PD-1^+^Foxp3^+^ Tfr was significantly reduced, leading to an inverse relationship between Tfh and Tfr (Fig. 1C, E, and I).

Consistent with murine findings, SLE patients exhibited elevated peripheral CXCR5^+^PD-1^+^ cell frequencies versus healthy controls (Fig. 1F and G). Subpopulation analysis defined Tfh (CD25^−^CD127^High^) and Tfr (CD25^+^CD127^Low^), demonstrating significant Tfh expansion and Tfr depletion in SLE patients (Fig. 1F, H, and I). Clinically, Tfh abundance exhibited a significant positive correlation with Systemic Lupus Erythematosus Disease Activity Index (SLEDAI) scores, while Tfr levels inversely correlated with disease activity (Fig. 1J). These results demonstrate an imbalance of Tfh/Tfr in SLE, indicating that the restoration of this imbalance could serve as a potential therapeutic target for SLE.

### MSC-Exos restrain the differentiation and function of Tfh both in vitro and in vivo

**Fig. 2.**
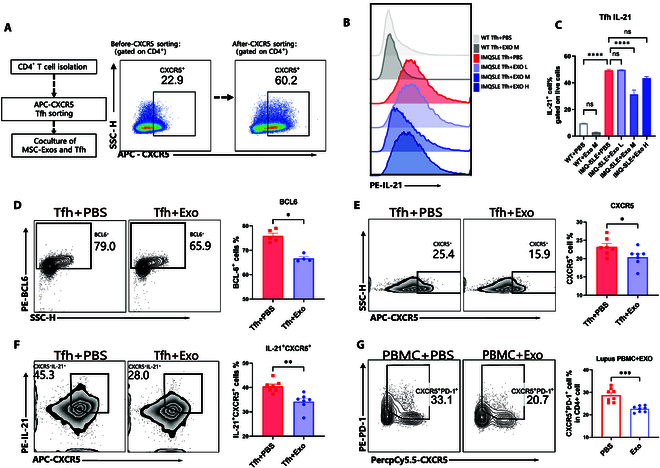
MSC-Exos inhibited the function of mature Tfh cells sorted from IMQ-SLE in vitro. (A) Isolation of mature Tfh in IMQ-SLE (*n* = 14). (B and C) Mature Tfh from IMQ-SLE (*n* = 14) were cocultured with varying concentrations of MSC-Exos for 24 h, and IL-21 secretion levels were detected using flow cytometry. (D) MSC-Exos were cocultured with mature Tfh from IMQ-SLE (*n* = 5) for 24 h, and BCL6 expression levels were measured using flow cytometry. (E) MSC-Exos were cocultured with mature Tfh from IMQ-SLE (*n* = 7) for 24 h, and CXCR5 expression levels were examined using flow cytometry. (F) MSC-Exos were cocultured with mature Tfh from IMQ-SLE (*n* = 7) for 24 h, and the proportion of IL-21^+^CXCR5^+^ cells was assessed using flow cytometry. (G) MSC-Exos were cocultured with PBMCs of SLE patients (*n* = 8) for 24 h, and the proportion of CD4^+^PD-1^+^CXCR5^+^ cells was evaluated using flow cytometry. Bars indicate the means ± SEM. ns, not significant; *P < 0.05; **P < 0.01; ***P < 0.001; ****P < 0.0001 determined by *t* test or one-way ANOVA with Tukey’s multiple comparisons test or 2-tailed unpaired Student’s *t* test.

In this study, we utilized exosomes derived from AD-MSCs, referred to as MSC-Exos. To assess the potential of MSC-Exos in inhibiting Tfh, we first conducted in vitro experiments. Using magnetic-activated cell sorting (MACS), we isolate CD4^+^ T cells from spleens and further enriched for Tfh by selecting those with high CXCR5 expression, achieving a purity exceeding 60%, indicative of functionally mature Tfh in IMQ-SLE (Fig. 2A). Subsequently, we cocultured these sorted Tfh with MSC-Exos at different concentrations for 24 h. The concentration gradient of MSC-Exos was as follows: low concentration: 0.8 × 10^8^ particles/ml; medium concentration: 1.6 × 10^8^ particles/ml; high concentration: 3.2 × 10^8^ particles/ml. We then examined IL-21 expression in sorted Tfh after coculture with MSC-Exos or PBS. Results showed that the medium concentration of MSC-Exos significantly suppressed IL-21 expression, while low and high concentrations showed no significant difference (Fig. 2B and C). Consequently, the moderate concentration of MSC-Exos was chosen for subsequent experiments (1.6 × 10^8^ particles/ml). We also assessed BCL6 expression in Tfh cocultured with MSC-Exos, which showed a significant reduction (Fig. 2D). CXCR5 also decreased notably, along with a decrease in CXCR5^+^IL-21^+^ double-positive cells (Fig. 2E and F). To determine if MSC-Exos have similar effects on Tfh from lupus patients, peripheral blood mononuclear cells (PBMCs) were isolated and cocultured with MSC-Exos. Flow cytometry indicated that MSC-Exos could also inhibit Tfh derived from lupus patients in vitro (Fig. 2G). These results indicated that MSC-Exos significantly suppress the function of Tfh in IMQ-SLE mice and lupus patients, as evidenced by reduced secretion of IL-21 by Tfh and decreased expression levels of BCL6 and CXCR5.

**Fig. 3.**
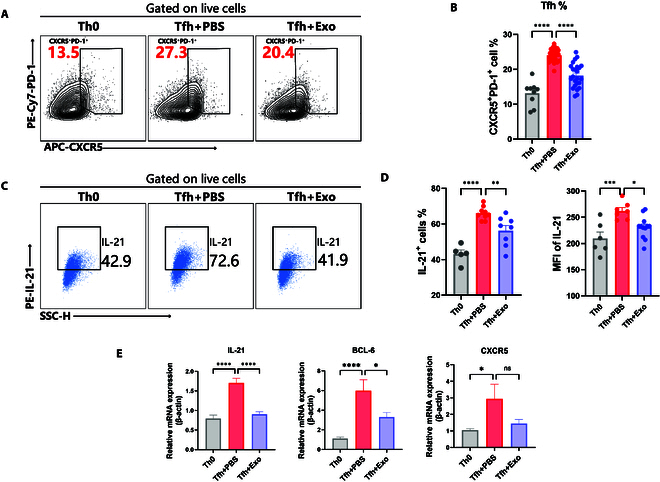
MSC-Exos suppressed Tfh differentiation in vitro. (A and B) Naïve CD4^+^ T cells were extracted from WT Balb/C mice and cultured under Tfh polarization conditions for 72 h with CD3/28 stimulation in vitro, with coculture of MSC-Exos or PBS as control. Th0 represents naïve CD4 T cells cultured under CD3/28 stimulation for 72 h, without Tfh polarization conditions. The proportion of CXCR5^+^PD-1^+^ Tfh cells was determined using flow cytometry. (C and D) IL-21 expression was analyzed in the Tfh-induced differentiation system after coculture with MSC-Exos or PBS by flow cytometry. (E) IL-21, BCL6, and CXCR5 mRNA expression levels were analyzed in the Tfh polarization condition system after coculture with MSC-Exos or PBS by RT-qPCR. Bars indicate the means ± SEM. *P < 0.05, **P < 0.01, ***P < 0.001, ****P < 0.0001 determined by one-way ANOVA with Tukey’s multiple comparisons test.

To investigate the impact of MSC-Exos on differentiation of Tfh, we induced Tfh under Tfh polarization condition in vitro. First, naïve CD4^+^ T cells were isolated followed by in vitro Tfh polarization for 72 h (Fig. S5 and S6). The condition for Tfh polarization included 50 ng/ml IL-6, 10 μg/ml anti-IFN-γ, 10 μg/ml anti-IL-4, 10 μg/ml anti-TGF-β, and 5 μg/ml anti-IL-2 [20,21]. Concurrently, naïve CD4^+^ T cells under nonpolarization conditions (Th0) were used as a negative control. After 72 h, Tfh expressing high levels of PD-1 and CXCR5 were stably induced in vitro (Fig. 3A). Based on previous results, we cocultured the Tfh with a medium concentration (1.6 × 10^8^ particles/ml) of MSC-Exos under polarization conditions to achieve optimal inhibitory effects. The results showed that MSC-Exos significantly reduced the proportion of CXCR5^+^PD-1^+^ Tfh and IL-21 expression in vitro (Fig. 3A to D). Furthermore, RT-qPCR analysis revealed that MSC-Exos significantly suppressed the elevated mRNA levels of IL-21 and BCL6 in the Tfh polarization system, while CXCR5 expression showed no statistically significant difference (Fig. 3E). These findings collectively indicate that MSC-Exos effectively suppress Tfh differentiation in vitro.

**Fig. 4.**
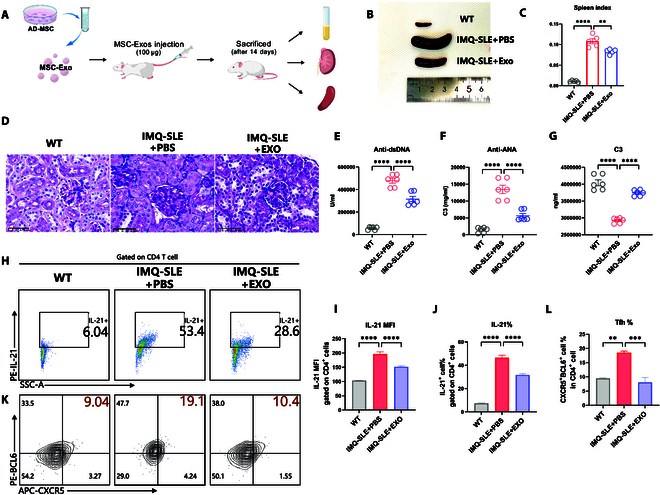
IMQ-SLE mice received an intravenous administration of 100 μg of MSC-Exos (2.25 × 10^9^ particles) and were sacrificed after 14 d. (A) Serum was collected to measure anti-dsDNA antibody, anti-ANA antibody, and C3 levels. Kidneys were harvested for periodic acid–Schiff (PAS) staining. Spleens were extracted to evaluate the inhibitory effect of MSC-Exos on Tfh in vivo. (B and C) Representative spleens from each group. MSC-Exos treatment inhibited spleen enlargement and reduced the spleen index (*n* = 6). (D) PAS staining of kidney samples. Scale bar, 50 μm. (E to G) MSC-Exos treatment reduced serum anti-dsDNA and anti-ANA antibody levels and increased serum C3 levels (*n* = 3). (H to J) Proportions and mean fluorescence intensity (MFI) of IL-21% cells in WT (*n* = 6), IMQ-SLE + PBS (*n* = 3), and IMQ-SLE + Exo (*n* = 6) mice. (K and L) Proportions of BCL6^+^CXCR5^+^ % cells in WT (*n* = 6), IMQ-SLE + PBS (*n* = 6), and IMQ-SLE + Exo (*n* = 6) mice. Bars indicate the means ± SEM. *P < 0.05, **P < 0.01, ***P < 0.001, ****P < 0.0001 determined by one-way ANOVA with Tukey’s multiple comparisons test.

To evaluate the in vivo impact of MSC-Exos on Tfh in the IMQ-SLE model, we intravenously administered 100 μg of MSC-Exos (2.25 × 10^9^ particles) to IMQ-SLE mice, with PBS-treated mice serving as controls. After 14 d, all mice were sacrificed for further analysis (Fig. 4A). The MSC-Exos-treated group exhibited a significantly lower spleen index compared to the PBS control group, suggesting a reduction in splenomegaly (Fig. 4B and C). Histological analysis of kidney sections showed that MSC-Exos reduced glomerular capillary intracellular proliferation, indicating potential reno-protective effects. (Fig. 4D). Serological analysis via ELISA demonstrated that MSC-Exos significantly decreased serum levels of anti-dsDNA and anti-nuclear autoantibodies (ANA) while concurrently increasing serum complement C3 levels compared to the PBS control group, suggesting an improvement in autoimmune pathology (Fig. 4E to G).

To further assess the impact of MSC-Exos on Tfh in vivo, CD4^+^ T cells were extracted from the spleens and Tfh populations were quantified using flow cytometry. Results indicated a notable reduction in IL-21 expression levels and a significant decrease in the proportion of CXCR5^+^BCL6^+^ cells among CD4^+^ T cells in the MSC-Exos-treated group, compared to the PBS-treated controls (Fig. 4H, I, K, and L).

Collectively, these findings indicate that MSC-Exos have significant therapeutic effects on IMQ SLE mice, and further suggest that MSC-Exos exert therapeutic effects on lupus mice by inhibiting Tfh.

### MSC-Exos regulate calcium homeostasis in Tfh via Stim1–Orai1 signaling

**Fig. 5.**
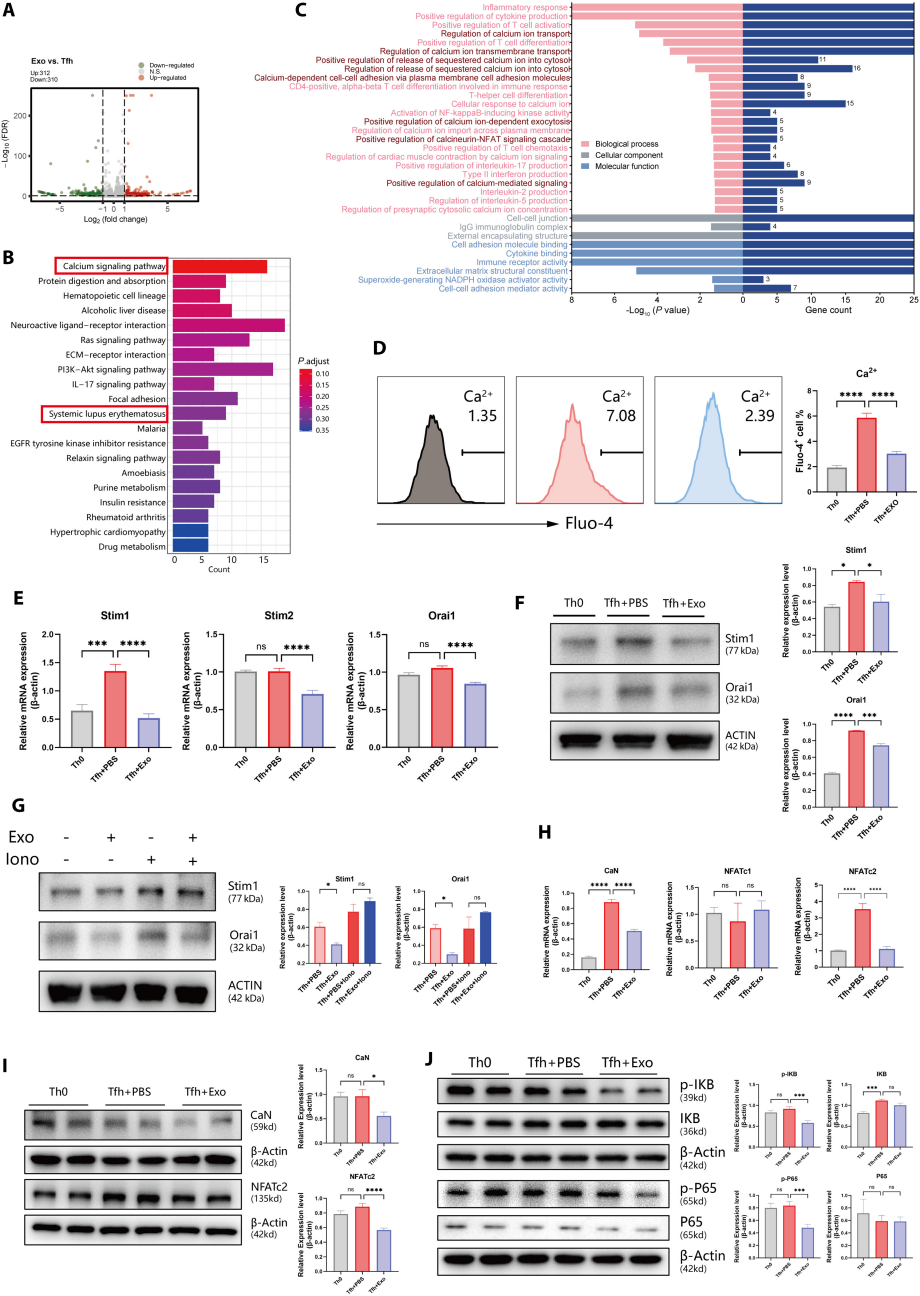
MSC-Exos regulate calcium homeostasis in Tfh. (A) Volcano diagram of DEGs of Tfh between control group and Exo group (*n* = 3). (B) KEGG enrichment analysis of Tfh between control group (*n* = 3) and Exo group (*n* = 3). These pathways were significant differences in the diagram. (C) GO enrichment analysis of DEGs between Tfh and naïve CD4^+^ T cells in C57 based on the GSE157648 dataset. (D) Fluo-4 AM-loaded Th0 and Tfh treated with/without MSC-Exos in HBSS medium for 30 min, and intracellular Ca^2+^ levels were detected by flow cytometry using Fluo-4 AM as the probe (*n* = 5). (E) mRNA expression levels of Stim1, Stim2, and Orai1 in Tfh with PBS or MSC-Exos coculture were detected by RT-qPCR. (F) WB analysis of Stim1 and Orai1 protein expression in Th0 and Tfh after treatment with MSC-Exos or PBS. (G) WB analysis of Stim1 and Orai1 protein expression in Tfh treated with/without MSC-Exos, and ionomycin (5 μM) was used to elevate intracellular Ca^2+^ levels. (H) RT-qPCR analysis of NFATc1, NFATc2, and CaN mRNA expression levels in Tfh after treatment with MSC-Exos or PBS. (I) WB analysis of CaN and NFATc2 protein expression in Tfh treated with/without MSC-Exos. (J) WB analysis of IκB, p-IκB, P65, and p-P65 protein expression in Tfh treated with/without MSC-Exos. Bars indicate the means ± SEM. *P < 0.05, **P < 0.01, ***P < 0.001, ****P < 0.0001 determined by one-way ANOVA with Tukey’s multiple comparisons test.

To further elucidate the specific mechanisms by which MSC-Exos regulate Tfh, we performed RNA-seq on MSC-Exos-treated Tfh cells, with PBS-treated cells serving as control. Differentially expressed gene (DEG) analysis followed by Kyoto Encyclopedia of Genes and Genomes (KEGG) pathway enrichment analysis revealed significant enrichment of the calcium signaling pathway, alongside notable enrichment of pathways associated with SLE (Fig. 5A and B). Additionally, Gene Ontology (GO) enrichment analysis of dataset GSE157648 from the Gene Expression Omnibus (GEO) database comparing Tfh to naïve CD4^+^ T cells demonstrated significant enrichment of calcium-related pathways (Fig. 5C). These findings suggest that the calcium signaling pathway is markedly activated in Tfh, and MSC-Exos may exert their regulatory effects on Tfh by modulating calcium homeostasis.

The calcium-related pathway plays a crucial role in CD4^+^ T cell-mediated autoimmune responses [22]. To investigate calcium dynamics in Tfh, we utilized Fluo-4 staining to measure intracellular Ca^2+^ concentrations. The results revealed a significant elevation in Ca^2+^ levels in Tfh compared to Th0. Notably, treatment with MSC-Exos markedly reduced intracellular calcium levels (Fig. 5D), suggesting a regulatory role of MSC-Exos in calcium homeostasis.

The Stim1–Orai1 signaling is a critical regulator of intracellular calcium homeostasis. Upon T cell receptor (TCR) stimulation, endoplasmic reticulum (ER) calcium stores are depleted, leading to the activation and translocation of Stim1 to ER–plasma membrane junctions [23]. Stim then binds to and opens Orai1 channels, facilitating extracellular calcium influx. To validate the regulatory effect of MSC-Exos on the Ca^2+^ upstream signaling, we first assessed the mRNA levels of the key initiators: Stim1, Stim2, and Orai1. RT-qPCR results demonstrated that Stim1 mRNA was significantly up-regulated in Tfh, while MSC-Exos treatment markedly reduced Stim1, Stim2, and Orai1 (Fig. 5E). These findings were further confirmed by WB analysis (Fig. 5F). Ionomycin, a commonly used stimulator of intracellular calcium signaling, was subsequently employed to further validate the effects of MSC-Exos as positive control. WB results revealed that the down-regulation of Stim1 and Orai1 by MSC-Exos was attenuated in the presence of ionomycin (Fig. 5G). Collectively, these results indicate that MSC-Exos can modulate the activation of the Ca^2+^ upstream signaling in Tfh through Stim1 and Orai1.

Elevated cytoplasmic calcium levels activate calcineurin (CaN), a serine/threonine phosphatase, which dephosphorylates and activates downstream targets—NFAT [22]. Dephosphorylated NFAT translocates to the nucleus, where it initiates transcription of genes essential for T cell proliferation, differentiation, and pro-inflammatory cytokine production [23]. We first confirmed the up-regulation of CaN and NFATc2 in lupus Tfh in public dataset GSE157648 (Fig. S7). To investigate the effects of MSC-Exos on the Ca^2+^ downstream signaling in Tfh, we quantified CaN and NFAT levels. Tfh exhibited significantly higher CaN and NFATc2 mRNA levels compared to Th0. MSC-Exos treatment reduced CaN expression by >50% and normalized NFATc2 to Th0 levels, whereas NFATc1 exhibited minimal variation (Fig. 5H). WB confirmed parallel reductions in CaN and NFATc2 protein levels induced by MSC-Exos (Fig. 5I).

Nuclear factor κB (NF-κB) is also located on the downstream of the calcium signaling pathway, sharing similar stimuli with NFAT by Ca^2+^ [24]. In resting T cells, the NF-κB subunit P65 (RelA) binds to inhibitory IκB proteins. TCR stimulation triggers IκB kinase (IKK)-mediated IκB phosphorylation, leading to its proteasomal degradation and subsequent P65 nuclear translocation to drive inflammatory gene expression [25]. This classical process in Tfh is responsible for up-regulating CXCR5, IL-21, and ICOS expression, contributing to Tfh differentiation and function [26]. Given the interplay between NFAT and NF-κB pathways in immune regulation [27], we further investigated NF-κB signaling. By analyzing the GSE31702 from GEO database, we confirmed the significant activation of the NF-κB pathway in Tfh from SLE compared to those from healthy controls (Fig. S7). To determine whether MSC-Exos also regulate on this downstream calcium signaling, we examined the associated protein expression. Tfh showed a significant increase in IκB protein levels compared to Th0. In contrast, coculturing with MSC-Exos led to a notable decrease in IκB phosphorylation levels (Fig. 5J). Similarly, the phosphorylation of P65 in Tfh significantly decreased after coculture with MSC-Exos, while total P65 protein levels remained unchanged (Fig. 5J). These results suggest that MSC-Exos stabilize IκB, thereby inhibiting the dissociation of P65 from IκB, reducing the activation level of P65 phosphorylation and preventing its nuclear translocation. Collectively, MSC-Exos exert an inhibitory effect on Tfh differentiation and function by regulating the Ca^2+^-mediated NFAT and NF-κB pathway.

### MSC-Exos alleviate mitochondrial damage by inhibiting MCU-induced calcium overload

**Fig. 6.**
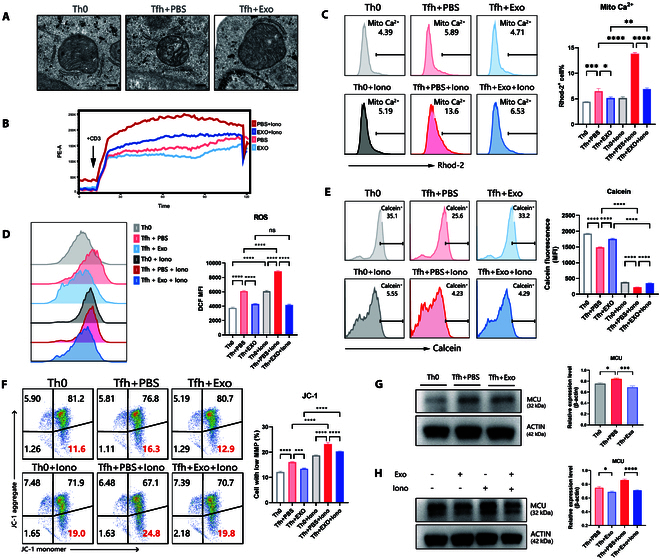
MSC-Exos alleviate mitochondrial damage in Tfh by inhibiting MCU-induced calcium overload. (A) Representative TEM images of mitochondria in Th0 and Tfh, with coculture of MSC-Exos or PBS as control. Scale bars, 200 nm. (B) Rhod-2 AM-loaded Th0 and Tfh treated with/without MSC-Exos in HBSS medium for 30 min, and mito-Ca^2+^ influx rates were detected by flow cytometry using Rhod-2 AM as the probe. Tfh was stimulated with anti-CD3-biotin (5 μg/ml). Ionomycin (5 μM) was used to increase mito-Ca^2+^ levels. (C) Measurements of mito-Ca^2+^ levels in Tfh after 60-s stimulation of anti-CD3-biotin (5 μg/ml) using Rhod-2 AM as the probe. Ionomycin (5 μM) was used to increase mito-Ca^2+^ levels (*n* = 10). (D) Representative flow cytometric analysis of ROS production in Tfh using DCFH-DA as the probe. Ionomycin (5 μM) was used to increase mito-Ca^2+^ levels (*n* = 10). (E) Representative flow cytometric analysis of mPTP opening levels in Tfh using calcein as the probe. The higher degree of mPTP opening, the lower calcein fluorescence intensity (*n* = 10). (F) JC-1 assay evaluation of the MMP level of Th0 and Tfh treated with/without MSC-Exos. Ionomycin (5 μM) was used to increase mito-Ca^2+^ levels (*n* = 10). (G and H) WB analysis of MCU protein expression in Tfh treated with/without MSC-Exos, and ionomycin (5 μM) was used to elevate intracellular Ca^2+^ levels. Bars indicate the means ± SEM. *P < 0.05, **P < 0.01, ***P < 0.001, ****P < 0.0001 determined by one-way ANOVA with Tukey’s multiple comparisons test.

Intracellular calcium homeostasis is crucial for the mitochondrial quality control. For mitochondria, calcium is a double-edged sword: Low levels are essential for optimal adenosine triphosphate (ATP) production, while calcium overload impairs its function [28]. We have demonstrated that Tfh exhibited disrupted calcium homeostasis, which was restored by MSC-Exos via inhibition of Stim1/Orai1-mediated calcium signaling. To further explore the consequences of calcium dysregulation, we employed electron microscopy, which revealed significant mitochondrial morphological abnormalities and increased autophagy in Tfh. Notably, these pathological changes were markedly alleviated by MSC-Exos treatment (Fig. 6A). Given the close association between mitochondria and calcium homeostasis, we further investigated the impact of MSC-Exos on mitochondrial calcium regulation.

First, we monitored the mitochondrial calcium influx rate using Rhod-2 AM, which is widely used to test the mitochondrial Ca^2+^ [29]. Results revealed that MSC-Exos treatment significantly suppressed the mitochondrial Ca^2+^ influx rate in Tfh (Fig. 6B). Additionally, MSC-Exos effectively inhibited the ionomycin-induced increase in mitochondrial Ca^2+^ influx rate, even in the ionomycin-treated group (Fig. 6B). Subsequent measurements showed that mitochondrial Ca^2+^ levels in Tfh were significantly elevated compared with Th0, and ionomycin further accentuated this difference. Notably, MSC-Exos effectively prevented mitochondrial calcium overload in Tfh in the presence and absence of ionomycin (Fig. 6C).

Mitochondrial calcium overload leads to a range of mitochondrial damage [28]. Given the pivotal role of mitochondria as cellular powerhouses, their dysfunction directly contributes to oxidative stress. To investigate this, we assessed ROS levels in Tfh. Flow cytometry analysis demonstrated that, as expected, Tfh exhibited significantly higher ROS levels than Th0. Notably, treatment with MSC-Exos markedly reduced ROS levels, even after ionomycin treatment—which further increases mitochondrial Ca^2+^—MSC-Exos continued to alleviate oxidative stress effectively (Fig. 6D). Furthermore, we evaluated the quality of mitochondria by examining the mitochondrial permeability transition pore (mPTP) and mitochondrial membrane potential (MMP, ΔΨm). Under normal conditions, mPTP remains closed, but stimuli such as oxidative stress or calcium overload induce its opening. The opening of the mPTP disrupts the mitochondrial membrane potential (MMP, ΔΨm) and consequently leads to mitochondrial dysregulation and impaired ATP production. We further evaluated the levels of mPTP opening and MMP using flow cytometry. Calcein was employed to assess the extent of mPTP opening, with a higher degree of mPTP opening corresponding to a lower calcein fluorescence intensity. JC-1 was employed to evaluate MMP. The results demonstrated that Tfh exhibited a higher degree of mPTP opening and a more pronounced reduction in MMP compared to Th0, and MSC-Exos effectively ameliorated these mitochondrial abnormalities (Fig. 6E and F). Moreover, in the presence of ionomycin to exacerbate mitochondrial calcium overload, Tfh suffered more severe mitochondrial damage—characterized by an even greater mPTP opening and further MMP declines—yet MSC-Exos maintained their protective effect against mitochondrial injury (Fig. 6E and F).

MCU, a selective calcium channel on the inner mitochondrial membrane, is a key contributor to mitochondrial calcium overload when its expression is dysregulated, and it has recently been linked to CD4^+^ T cell activation [30]. In line with our findings on mitochondrial damage in Tfh, we next examined MCU protein expression to elucidate how MSC-Exos modulate mitochondrial calcium overload. WB analysis revealed that MCU expression was significantly up-regulated in Tfh compared with Th0, and treatment with MSC-Exos markedly normalized this aberrant expression. Furthermore, when ionomycin was applied to exacerbate calcium overload, MCU overexpression in Tfh was further amplified; however, MSC-Exos continued to significantly reduce MCU levels.

Collectively, these findings demonstrate that mitochondrial calcium overload in Tfh induces mitochondrial damage, as evidenced by ROS burst, abnormal mPTP opening, and reduced MMP, and MSC-Exos effectively mitigate this overload through modulation of MCU, thereby suppressing Tfh differentiation and function.

### MSC-Exos restore the imbalance of Tfh/Tfr in SLE

During the validation of the effects of MSC-Exos on Tfh differentiation, we observed that although MSC-Exos significantly suppressed IL-21 and BCL6 expression and markedly reduced CXCR5^+^PD-1^+^ Tfh population, their inhibitory effect on CXCR5 mRNA expression was not apparent (Fig. 3E). Given that Tfr and Tfh share the surface marker CXCR5 and that the restoration of the Tfr/Tfh imbalance is closely associated with the amelioration of SLE [31], we hypothesized that MSC-Exos may concurrently inhibit Tfh while promoting Tfr expansion and function.

**Fig. 7.**
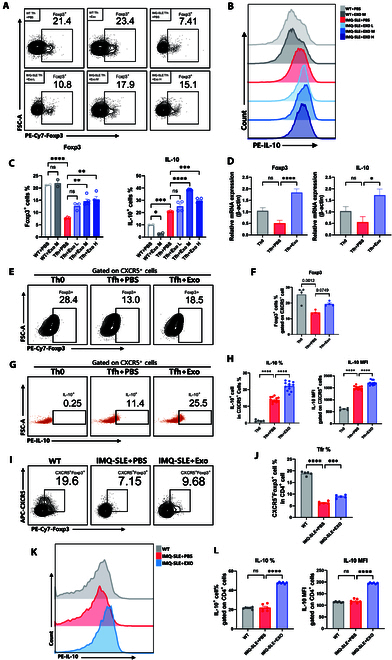
MSC-Exos suppressed Tfh while promoting the differentiation and function of Tfr. (A to C) Isolated CXCR5^+^ cells from IMQ-SLE (*n* = 4) were cocultured with gradient concentrations of MSC-Exos in vitro for 24 h, and Foxp3 and IL-10 expression levels were assessed by flow cytometry. (D) The mRNA expression levels of Foxp3 and IL-10 in the Tfh polarization conditions were measured using RT-qPCR. (E to H) The expression levels of Foxp3 and IL-10 were detected in CXCR5^+^ cells within Tfh polarization conditions by flow cytometry. (I to L) Proportions of CD4^+^CXCR5^+^Foxp3^+^ cells and CD4^+^IL-10^+^ cells in WT (*n* = 5), IMQ-SLE + PBS (*n* = 5), and IMQ-SLE + Exo (*n* = 5) mice. Bars indicate the means ± SEM. *P < 0.05, **P < 0.01, ***P < 0.001, ****P < 0.0001 determined by one-way ANOVA with Tukey’s multiple comparisons test.

To test this, we firstly isolated CD4^+^CXCR5^+^ cells—which comprise both mature Tfh and Tfr populations—from the spleens of IMQ-SLE mice and cocultured them with MSC-Exos at the same 3 concentrations used in previous experiments. Flow cytometry analysis revealed that IMQ-SLE-derived CXCR5^+^ cells exhibited significantly reduced levels of Foxp3, a key marker of Tfr, compared with controls (Fig. 7A and C). Both moderate and high concentrations of MSC-Exos significantly increased the proportions of Foxp3^+^ and IL-10^+^ cells, indicating enhanced Tfr functionality (Fig. 7B and C). Notably, the effect on IL-10 expression was most pronounced at the moderate MSC-Exos concentration (Fig. 7B and C), mirroring our earlier observations regarding IL-21 suppression (Fig. 2B and C). Specifically, MSC-Exos at moderate concentrations significantly inhibited Tfh functionality while concurrently most effectively promoting Tfr function. Additionally, we also evaluated the influence of MSC-Exos on the differentiation of Tfr in vitro. RT-qPCR and flow cytometry results confirmed that MSC-Exos significantly increased Foxp3 and IL-10 levels in CXCR5^+^ cells under Tfh polarization condition (Fig. 7D to H).

Extending our findings in vivo, we further investigated the effects of MSC-Exos on Tfr in IMQ-SLE mice. The results revealed a significant decrease in the frequency of CD4^+^CXCR5^+^Foxp3^+^ Tfr in IMQ-SLE compared to WT mice, which was markedly restored following intravenous injection of 100 μg of MSC-Exos (Fig. 7I and J). Moreover, MSC-Exos treatment significantly elevated IL-10 expression levels in IMQ-SLE mice (Fig. 7K and L).

Taken together, these results demonstrate that MSC-Exos exert a bidirectional regulatory effect on the Tfh/Tfr balance—suppressing Tfh differentiation and function while simultaneously enhancing Tfr expansion and functionality. This dual action may underlie the therapeutic potential of MSC-Exos in ameliorating SLE.

## Discussion

**Fig. 8.**
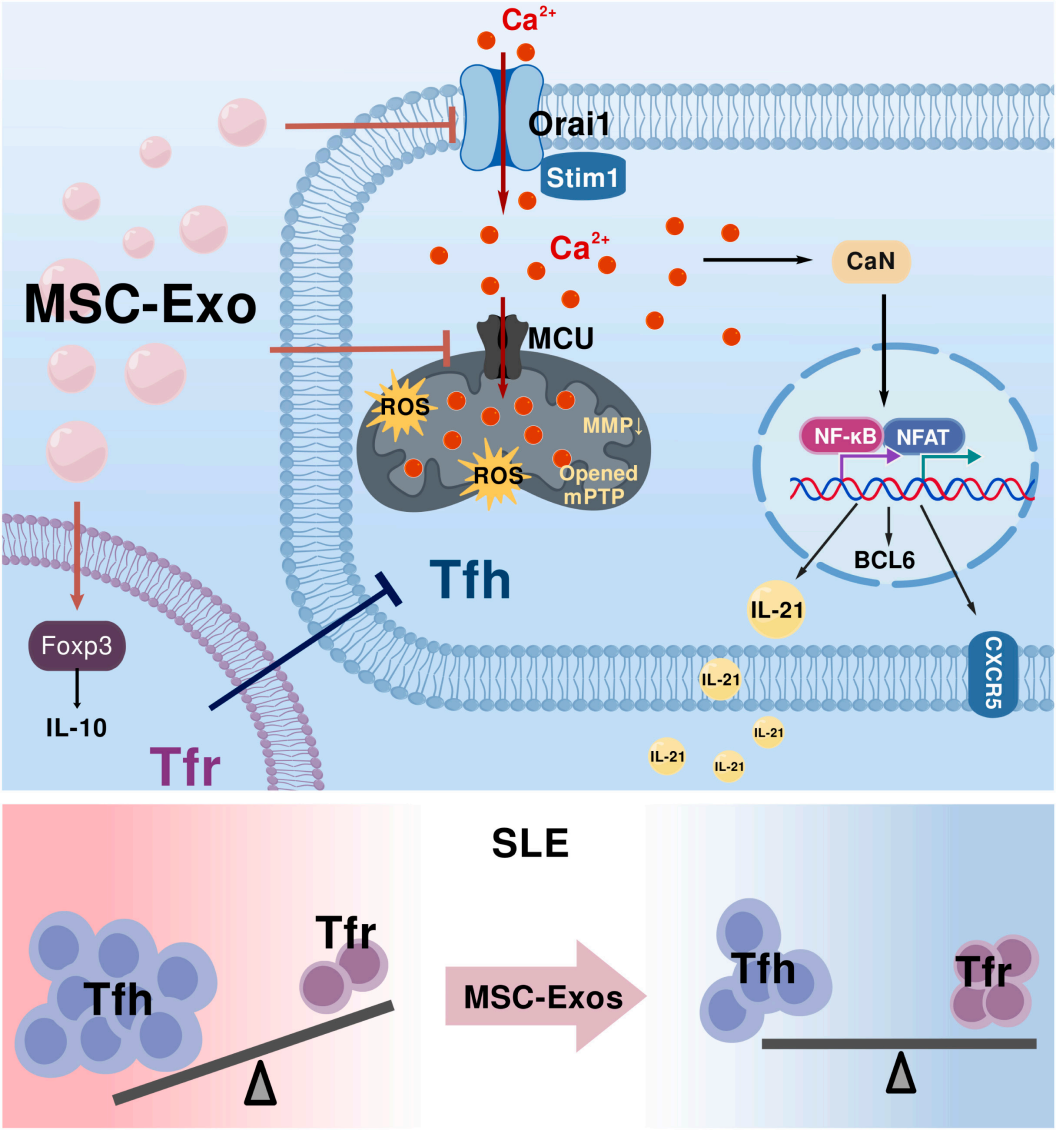
MSC-Exos regulate Tfh differentiation and function via a multi-target network, restoring the Tfh/Tfr balance and alleviating SLE. Mechanistically, MSC-Exos inhibit Tfh activation by correcting intracellular calcium dysregulation and preventing mitochondrial calcium overload. In the cytoplasm, MSC-Exos restrict calcium influx through inhibition of the Stim1/Orai1 expression, thereby reducing NFAT and NF-κB activation. In the mitochondria, MSC-Exos suppress aberrant MCU expression, mitigating calcium overload-induced mitochondrial damage, and restoring mitochondrial homeostasis in Tfh. Figure was created with BioGDP [50].

SLE is a chronic autoimmune disease driven by autoantibody production, leading to multi-organ damage and increased mortality [32]. Tfh are crucial in assisting B cells to produce high-affinity antibodies and are highly activated and proliferative in SLE [2]. Given the importance of Tfh in lupus pathogenesis and the therapeutic potential of MSC-Exos in lupus [13,33], we investigated the effect and mechanism of MSC-Exos on Tfh in SLE. Our findings demonstrate that MSC-Exos effectively inhibit Tfh function and differentiation by restoring calcium homeostasis through Stim1–Orai1 pathway and calcium overload-induced mitochondrial damage. Moreover, we demonstrated for the first time the bidirectional regulatory effect of MSC-Exos on Tfh/Tfr balance in SLE (Fig. 8). Interestingly, we observed a nonlinear dose–response, with medium concentrations of MSC-Exos showing greater efficacy than higher doses. This may reflect cytokine overload, uptake saturation, or adaptive resistance within the inflamed lupus microenvironment.

The hyperactivation of the TLR7 signaling pathway is a key factor in the pathogenesis of lupus [34]. In this study, we established a lupus model in mice using IMQ, a TLR7 agonist, which is widely used in lupus research [17]. Female mice were used to reflect the female-biased prevalence of SLE and to remain consistent with commonly used lupus models [33]. We confirmed that IMQ-SLE mice and SLE patients had higher frequency of Tfh, along with a lower number of Tfr, indicating an imbalance in the Tfh/Tfr, consistent with other lupus models and patients [14,35]. Several studies have demonstrated the ability of MSCs to inhibit the activation of T cells in lupus [3]. MSC-Exos, which carry MSC immunoregulatory capabilities, have also shown great therapeutic value in autoimmune diseases [13]. However, whether MSC-Exos can effectively target Tfh and their potential underlying mechanisms remain unclear. In this study, we revealed that MSC-Exos suppress the excessive frequency of Tfh in SLE both in vivo and in vitro. Meanwhile, MSC-Exos promote the population and function of Tfr, thereby regulating the Tfh/Tfr imbalance in SLE. Mechanically, MSC-Exos effectively regulate the calcium homeostasis in Tfh in both cytoplasm and mitochondria. For cytoplasm, MSC-Exos can target the Stim1–Orai1–NFAT/NF-κB pathway to inhibit Tfh differentiation and function. For mitochondria, MSC-Exos alleviate mitochondrial damage in Tfh by inhibiting MCU-induced calcium overload.

Dysregulation of calcium homeostasis leads to excessive T cell activation and proliferation, thereby exacerbating autoimmune responses. Stim1–Orai1 signaling is the key regulator of cellular calcium homeostasis. Stim1 and Orai1 control the initiation of calcium influx upstream, where Stim1 functions as the ER calcium sensor and Orai1 serves as the plasma membrane calcium channel [24]. Studies have demonstrated significantly elevated expression of Stim2 and Orai1 in lupus nephritis, and intervention with Orai1-specific small interfering RNA (siRNA) has been shown to markedly alleviate renal damage [36]. Vaeth et al. [37] revealed that knockdown of either Stim1 or Stim2 significantly impaired Tfh function by suppressing NFAT-mediated BCL6 transcription. In this study, MSC-Exos down-regulated Stim1/Orai1 expression, thereby modulating calcium influx upstream and ameliorating calcium homeostasis imbalance in Tfh.

In the downstream of calcium influx, MSC-Exos effectively blocked NFAT and NF-κB pathway activity in Tfh, which play crucial roles in regulating T cell activation and differentiation [38]. The NFAT pathway is commonly activated in SLE [23], with an observed up-regulation of CaN-dependent NFATc2 in CD4^+^ T cells, leading to enhanced inflammatory responses. However, the specific CD4^+^ T cell subset involved remains unclear [39]. It is speculated that elevated expression of NFAT in Th17 cells in lupus may contribute to the up-regulation of CD40L expression, exacerbating lupus symptoms [23]. Inhibiting the NFAT pathway has significant therapeutic value in SLE, with drugs like cyclosporine A and tacrolimus offering protection against lupus nephritis [40]. Evidences have shown that NFATc2 facilitates the maintenance of Tfh [37], and NFATc2 directly influences IL-21 and CD40L expression [41]. Additionally, tacrolimus notably suppresses Tfh in transplant recipients, mitigating rejection reactions [42]. These findings suggest that inhibiting the NFAT pathway in Tfh may significantly suppress immune responses in SLE, which is consistent with our observations. Similarly, the NF-κB pathway, a central regulator of inflammatory responses, is critical for Tfh function. NF-κB deficiency results in impaired CXCR5 expression [43], and NF-κB directly regulates IL-21 transcription, underscoring its importance in Tfh differentiation and function [26]. Here, we showed that MSC-Exos markedly inhibit the NF-κB pathway in Tfh, consistent with a recent evidence showing the anti-inflammatory effects of bone marrow-derived MSCs derived exosomes (BMSC-Exos) through NF-κB down-regulation to reduce intervertebral disc degeneration [44]. Furthermore, NFATc2 shares 142 common immune response gene targets with P65, allowing dynamic integration of the NFAT and NF-κB pathways in regulating T cell immune responses [27], which aligns with our research findings that MSC-Exos modulate both NFAT and NF-κB pathways in Tfh.

MCU-mediated mitochondrial calcium homeostasis is critical for various cellular processes, including energy metabolism and cell survival [45]. Dysregulation of MCU expression leads to mitochondrial calcium overload by facilitating Ca^2+^ influx from the cytosol to the mitochondrial matrix [30]. This overload impairs mitochondrial function primarily by inhibiting ATP hydrolase activity [28] and subsequently induces mitochondrial swelling and membrane rupture, triggering ROS burst and mPTP opening, ultimately resulting in downstream inflammatory cascades [30]. T cell-specific MCU knockout in an experimental autoimmune encephalomyelitis (EAE) model alleviated neuroinflammation by suppressing mitochondrial calcium overload, suggesting that MCU is a promising therapeutic target for autoimmune diseases [30]. Our findings indicate that calcium dysregulation not only initiates NFAT/NF-κB activation but also drives MCU-dependent mitochondrial calcium overload. Targeting the Stim/Orai1–MCU axis may therefore represent a promising strategy for SLE. In this study, MSC-Exos effectively counteract mitochondrial calcium overload via MCU in Tfh, even under ionomycin-induced exaggerated calcium overload conditions. Moreover, MSC-Exos significantly alleviate mitochondrial oxidative damage and restore mitochondrial homeostasis in Tfh, thereby exerting anti-inflammatory effects. These findings reveal that the regulation of Tfh by MSC-Exos in this study is not confined to a single target but involves simultaneous network regulation of Stim1–Orai1–NFAT/NF-κB axis and MCU-dependent mitochondrial calcium overload, resulting in potent inhibition of Tfh in SLE.

Moreover, we found that MSC-Exos not only suppress Tfh but also promote the differentiation and function of Tfr. NFATc1 is expressed in resting T cells, while NFATc2 expression can be up-regulated by NFATc1 upon T cell stimulation, creating a positive feedback loop that amplifies NFAT pathway [22]. Foxp3, a key transcription factor in Tregs, can bind to the P1 region of the NFATc2 promoter, inhibiting its interaction with NFATc1 and thereby suppressing NFATc2 production [46]. Our data suggest that MSC-Exos primarily target NFATc2 rather than NFATc1, likely through selective up-regulation of Foxp3, which directly inhibits NFATc2 transcription independently of NFATc1. However, this finding appears to contrast with studies showing that NFATc2 deficiency impairs Tfr cell numbers and function, exacerbating autoimmune responses [37]. This discrepancy contradicts our findings, suggesting that MSC-Exos may intervene in the Tfh/Tfr balance through multi-target mechanisms. For instance, the promotion of Tfr cells could be mediated by direct enhancement of Foxp3 expression or through alternative pathways, which warrants further investigation. Additionally, Foxp3 up-regulation may also contribute to the suppression of Tfh, as Foxp3 directly inhibits the activity of NF-κB and NFAT transcription factors in T helper cells [47]. This inhibitory effect is well documented in Tregs and certain tumor cells, where the Foxp3–NF-κB axis plays a critical role in Treg differentiation and function [48,49]. Notably, Foxp3 deficiency is associated with heightened NF-κB activity [49]. These findings suggest that, beyond modulating calcium homeostasis to inhibit NFAT and NF-κB pathways, MSC-Exos may further enhance their suppressive effects on Tfh by inducing Foxp3. Therefore, we hypothesis that MSC-Exos achieve dual regulation of Tfh and Tfr by promoting Foxp3 expression, which simultaneously suppresses Tfh hyperactivation and enhances Tfr-mediated immune regulation.

The limitations of this study should be noted. Firstly, we did not use lentivirus or gene knockout mice for reverse validation, mainly due to the scarcity and instability of Tfh and Tfr in vitro. Secondly, the application of MSC-Exos in mice lasted only 14 d, necessitating further investigation into the long-term effects of MSC-Exos. Additionally, the lack of a mature in vitro polarization condition system for Tfr hampers a comprehensive understanding of the specific mechanisms through which MSC-Exos regulate Tfr. Lastly, while our data establish MSC-Exos as an effective corrector of Tfh/Tfr imbalance in SLE, this mechanism may extend to other inflammatory conditions where Tfh/Tfr dysregulation serves as an indicator of autoimmune pathology severity.

## Conclusion

Our study elucidates that MSC-Exos significantly inhibit Tfh differentiation and function in SLE. Mechanistically, MSC-Exos inhibit Tfh activation by correcting intracellular calcium dysregulation and preventing mitochondrial calcium overload. In the cytoplasm, MSC-Exos restrict calcium influx through inhibition of the Stim1/Orai1 signaling, thereby reducing NFAT and NF-κB activation. In the mitochondria, MSC-Exos prevent mitochondrial calcium overload by down-regulating MCU, reducing mitochondrial damage in Tfh. Additionally, we demonstrate the bidirectional regulatory effect of MSC-Exos on Tfh/Tfr balance in SLE. These results not only highlight the therapeutic potential of MSC-Exos in SLE but also underscore the critical role of calcium homeostasis in SLE management.

## Ethical Approval

Ethical approval was obtained from the Ethics Committee of Zhongshan Hospital, Fudan University, Shanghai, China (approval no. B2023-0862R).

## Data Availability

The RNA-seq data of this study have been deposited in the CNGB Sequence Archive (CNSA) of the China National GeneBank DataBase (CNGBdb) under the accession number CNP0006973.
