# Supplementary material for: Mesenchymal Stem Cell-Derived Exosomes Inhibit Stim1–Orai1 Signaling and Calcium Overload-Induced Mitochondrial Damage of Follicular Helper T Cells in Lupus
Source: Biomater Res. 2025 Sep 22;29:0255. doi: 10.34133/bmr.0255 (PMC12451110; doi:10.34133/bmr.0255)
Supplement: Supplementary 1 — Figs.S1 to S7 Table S1 [file bmr.0255.f1.pdf]

Supplementary materials

Mesenchymal Stem Cell-Derived Exosomes Inhibit Stim1-Orai1 signaling and Calcium Overload-induced Mitochondrial Damage of Follicular Helper T Cells in Lupus

Yingyu Wang[1,2†], Qingyong Xiang[3†], Yueren Wu[4,5†], Xiaoyun Zhang[1,2], Zhongzhou Huang[6], Yan Wang[7], Ji Yang[8], Hejian Zou[1,2*], Xue Yang[1,2*]

1 Division of Rheumatology, Huashan Hospital, Fudan University, Shanghai, China
2 Institute of Rheumatology, Immunology and Allergy, Fudan University, Shanghai, China
3 Department of Rheumatology, Shanghai Fifth Peoples Hospital, Fudan University, Shanghai, China
4 Department of Integrative Medicine, Huashan Hospital, Fudan University, Shanghai, China
5 Institutes of Integrative Medicine, Fudan University, Shanghai, China
6 Department of Dermatology, Sun Yat-sen Memorial Hospital, Sun Yat-sen University, Guangzhou, China.
7 Central Lab, Huashan Hospital, Fudan University, Shanghai, China
8 Department of Dermatology, Zhongshan Hospital, Fudan University, Shanghai, China

† These authors contributed equally: Wang Yingyu, Xiang Qingyong, Wu Yueren.
* Corresponding Author
Hejian zou
Email: hjzou@fudan.edu.cn
Xue Yang
Email: xyang@unirheuma.org

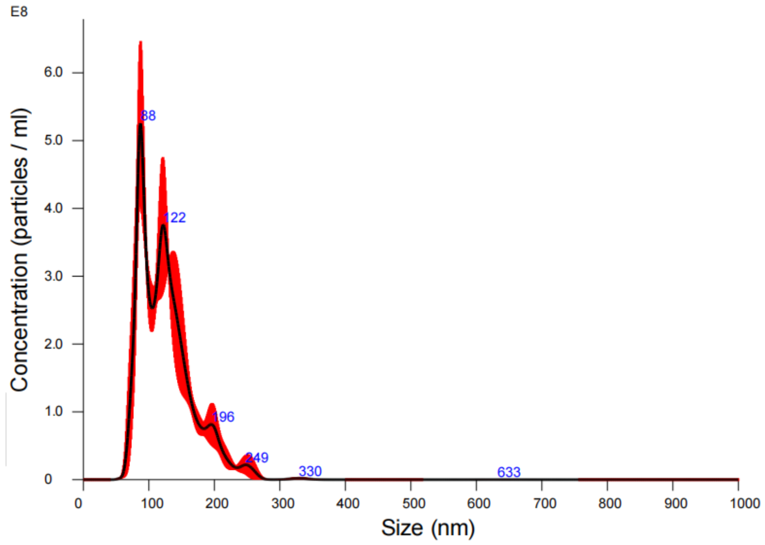

Supplementary Fig.1 Nanoparticle tracking analysis (NTA) was applied to detect the size distribution and particle concentration of MSC-Exos using a Nanosight NS300 instrument.

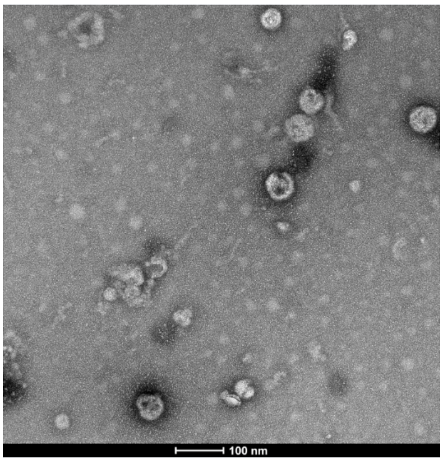

Supplementary Fig.2 Transmission electron microscopy (TEM) was utilized to examine the morphology of AD-MSC exosomes (MSC-Exos)

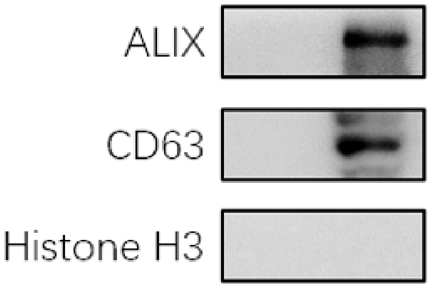

Supplementary Fig.3 Western blotting was utilized to confirmed the expression of surface markers CD63 and Alix, and the negative marker Histone 3.

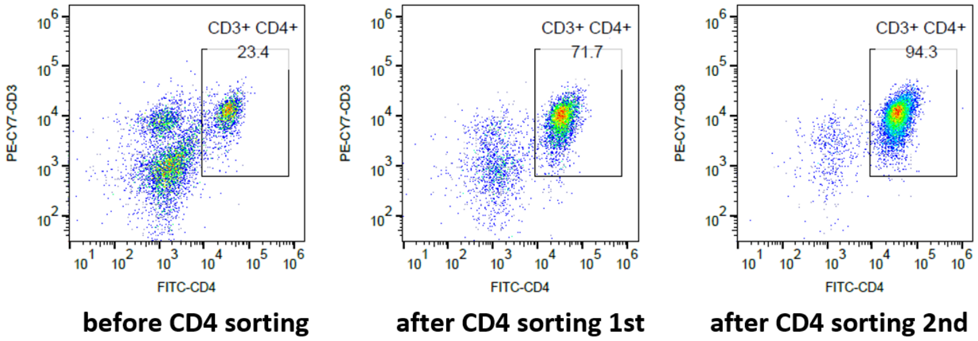

Supplementary Fig.4 The purity of sorted CD3$^+$CD4$^+$ T cells from IMQ-SLE spleens.

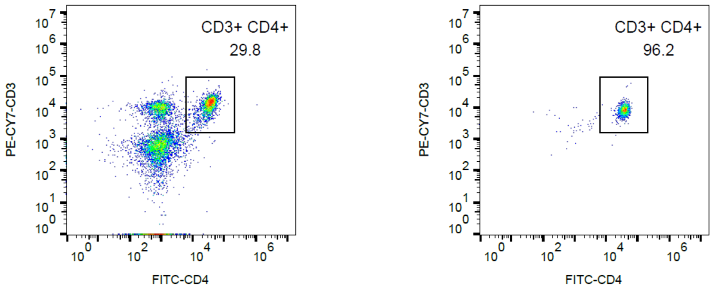

Supplementary Fig.5 The purity of sorted CD3$^+$CD4$^+$ T cells from WT spleens.

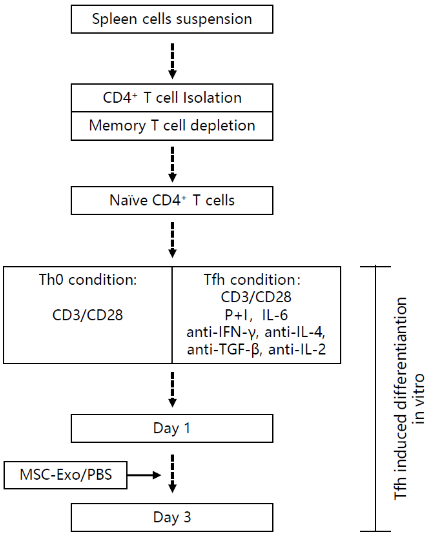

50 ng/mL IL-6 (10ug/ml)
10 μg/mL anti-IFN-γ (10mg/ml)
10 μg/mL anti-IL-4(2mg/ml)
10 μg/mL anti-TGFβ(1mg/ml)
5 μg/mL anti-IL-2(8mg/ml)

Supplementary Fig.6 Tfh polarization condition.

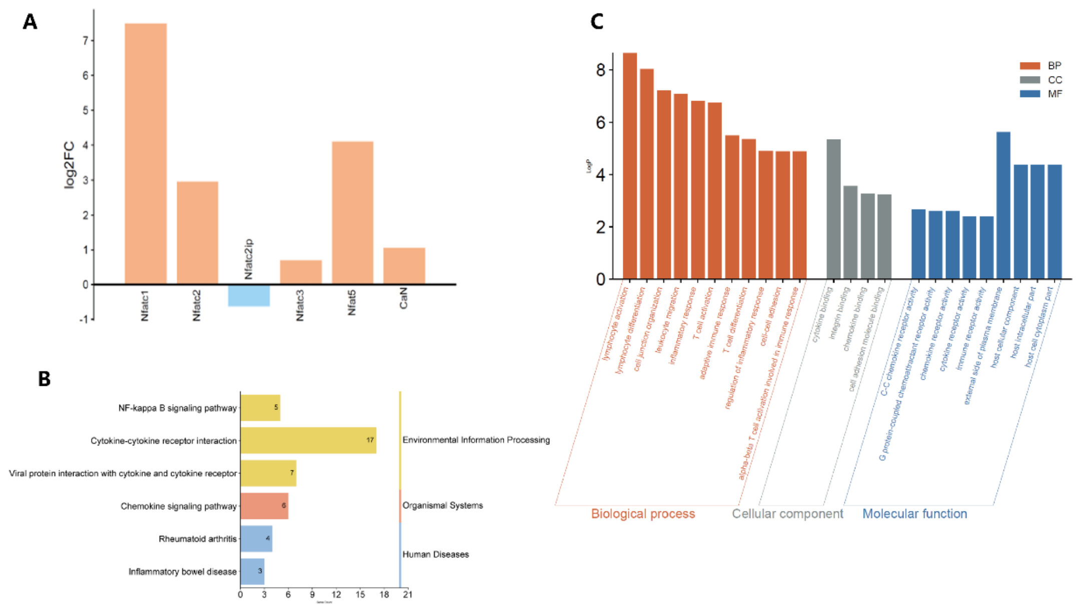

Supplementary Fig.7 Bioinformatic analysis of Tfh public dataset from GEO database. (A) DEG analysis of NFAT family and CaN in Tfh from lupus mice compared to healthy controls based on the GSE31702 dataset. (B) KEGG analysis of DEGs in Tfh from lupus mice compared to healthy controls based on the GSE31702 dataset. (C) GO enrichment of DEGs in Tfh from lupus mice compared to healthy controls based on the GSE31702 dataset.

Supplementary Table1. Mouse primer sequences.

gene name	primer sequences (from 5'-3')
mouse *Bcl6*	forward: CCTGAGGGAAGGCAATATCA
mouse *Bcl6*	reverse: CGGCTGTTCAGGAACTCTTC
mouse *Il21*	forward: CGCCTCCTGATTAGACTTCG
mouse *Il21*	reverse: GCCCCTTTACATCTTGTGGA
mouse *Cxcr5*	forward: TGGCCTTCTACAGTAACAGCA
mouse *Cxcr5*	reverse: GCATGAATACCGCCTTAAAGGAC
mouse *Il-10*	forward: TGCCAAGCCTTATCGGAAATGATCC
mouse *Il-10*	reverse: AGCCGCATCCTGAGGGTCTTC
mouse *Foxp3*	forward: TTTCACCTATGCCACCCTTATC
mouse *Foxp3*	reverse: GTAGGCGAACATGCGAGTAA
mouse *CaN*	forward: GTGAAAGCCGTTCCATTTCCA
mouse *CaN*	reverse: GAATCGAAGCACCCTCTGTTATT
mouse *Nfatc1*	forward: GGAGAGTCCGAGAATCGAGAT

mouse *Nfatc1*	reverse: TTGCAGCTAGGAAGTACGTCT
mouse *Nfatc2*	forward: TCATCCAACAACAGACTGCCC
mouse *Nfatc2*	reverse: GGGAGGGAGGTCCTGAAAACT
mouse *Stim1*	forward: GGCGTGGAAATCATCAGAAGT
mouse *Stim1*	reverse: TCAGTACAGTCCCTGTCATGG
mouse *Stim2*	forward: CTTGCGAGAACGGCTTTTTCG
mouse *Stim2*	reverse: GTACAGAGAGGAGGTGAGACTG
mouse *Orai1*	forward: GATCGGCCAGAGTTACTCCG
mouse *Orai1*	reverse: TGGGTAGTCATGGTCTGTGTC
